# The Impact of Diabetes Mellitus‐Related Oxidative Stress on Male Fertility: A Review

**DOI:** 10.1111/1753-0407.70157

**Published:** 2025-10-22

**Authors:** Simon Mwaringa Dena, Adesola Oluwaseun Adeleye, Kutullo Mohlala, Bridget Cebisile Langa, Chinyerum Sylvia Opuwari

**Affiliations:** ^1^ Department of Medical Biosciences, Faculty of Natural Sciences University of the Western Cape Cape Town South Africa

**Keywords:** diabetes mellitus, male infertility, oxidative stress, reactive oxygen species

## Abstract

Diabetes mellitus (DM) significantly impairs male reproductive health, largely through hyperglycemia‐induced oxidative stress (OS). Elevated glucose activates detrimental metabolic pathways, notably the polyol pathway, which depletes antioxidant defenses and generates reactive oxygen species (ROS). This oxidative burden damages spermatozoa, leading to reduced motility, abnormal morphology, DNA fragmentation, and disrupted membrane integrity. OS also compromises the hypothalamic–pituitary–gonadal axis, lowering testosterone synthesis and impairing spermatogenesis. The formation of advanced glycation end products (AGEs) and chronic inflammation further exacerbate Leydig and Sertoli cell dysfunction, microvascular injury, and testicular apoptosis. Clinical evidence consistently links DM to deteriorated semen parameters, hormonal imbalances, and reduced natural conception rates, with poorer outcomes in assisted reproductive technologies. Obesity and metabolic syndrome, common comorbidities in DM, amplify oxidative stress and further impair fertility potential. While seminal plasma contains enzymatic and non‐enzymatic antioxidants, these defenses are often insufficient in diabetic men. Targeted interventions, including antioxidant therapy, lifestyle modifications, glycemic control, and management of comorbidities, offer promise in mitigating oxidative damage. This review synthesizes current evidence on the molecular, endocrine, and clinical consequences of DM‐related oxidative stress on male fertility, underscoring the need for integrated management strategies to preserve reproductive function in diabetic men.

## Graphical abstract

The impact of Diabetes mellitus on male fertility.

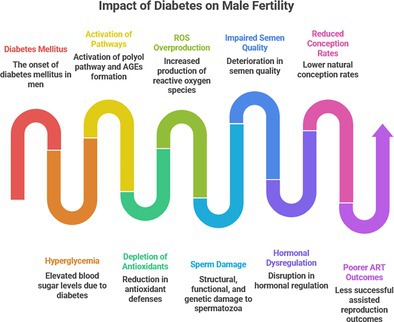

**Summary**

- Diabetes mellitus reduces male fertility by inducing oxidative stress that damages sperm quality and hormonal balance.
- Hyperglycemia activates harmful pathways, including the polyol pathway and advanced glycation end products (AGEs) formation, leading to testicular dysfunction.
- Clinical studies show diabetic men have poorer semen parameters, disrupted endocrine profiles, and reduced natural and ART success rates.
- Antioxidant therapy, lifestyle modifications, and metabolic control are potential strategies to mitigate oxidative damage and preserve fertility.

## 1 Background

Diabetes mellitus (DM) is a chronic metabolic disorder characterized by persistent hyperglycemia due to impaired insulin secretion, insulin action, or both [1]. Globally, an estimated 537 million adults were living with diabetes in 2021, with projections rising to 643 million by 2030 and 783 million by 2045 [2]. The condition is classified into four main types: type 1 diabetes mellitus (T1DM), an autoimmune destruction of pancreatic β‐cells; type 2 diabetes mellitus (T2DM), marked by progressive β‐cell dysfunction and insulin resistance; gestational diabetes mellitus (GDM), first recognized during pregnancy; and rarer forms caused by genetic defects or secondary to other diseases [3, 4, 5].

Male infertility defined as the inability to achieve pregnancy with a fertile partner after 12 months of regular, unprotected intercourse, is now recognized as a significant public health concern, affecting roughly 1 in 10 couples of reproductive age [6]. Male factors contribute solely to 30%–50% of infertility cases, and in combination with female factors to up to 30% of cases [7, 8]. The Global Burden of Disease Study 2019 estimated 56.5 million men affected worldwide, with the highest burden in the 30–34‐year age group [9, 10].

Mounting evidence links DM to male infertility, with oxidative stress (OS) emerging as a central pathogenic mechanism [11]. OS results from an imbalance between the excessive generation of reactive oxygen species (ROS) and the body's antioxidant defense capacity [12]. Spermatozoa are particularly susceptible to oxidative damage because of their high polyunsaturated fatty acid content and limited cytoplasmic antioxidant reserves [13]. Excess ROS can impair male reproductive function through multiple pathways including lipid peroxidation and loss of membrane fluidity, mitochondrial dysfunction, sperm DNA fragmentation, and disruption of the hypothalamic–pituitary–gonadal (HPG) axis, leading to reduced testosterone synthesis and impaired spermatogenesis [14, 15, 16]. Within the testes, OS damages both Leydig and Sertoli cells, exacerbates microvascular injury, and triggers germ cell apoptosis [17].

In DM, chronic hyperglycemia exacerbates OS through the activation of the polyol pathway [18]. The polyol pathway is a two‐step metabolic process that plays a significant role in glucose metabolism under hyperglycemic conditions [19]. The first step is catalyzed by aldose reductase (AR), which reduces glucose to sorbitol using nicotinamide adenine dinucleotide phosphate (NADPH) as a cofactor [20]. Subsequently, sorbitol dehydrogenase (SDH) utilizes nicotinamide adenine dinucleotide (NAD^+^) as a cofactor to facilitate the conversion of sorbitol to fructose [21]. This pathway becomes increasingly active during hyperglycemia, contributing to oxidative stress through three mechanisms. AR utilizes NADPH as a cofactor to reduce glucose to sorbitol. During hyperglycemia, up to 30% of glucose is shunted into the polyol pathway, leading to significant depletion of NADPH [22]. This depletion compromises the activity of glutathione reductase in regenerating reduced glutathione (GSH) levels and overall antioxidant capacity [23].

**FIGURE 1:**
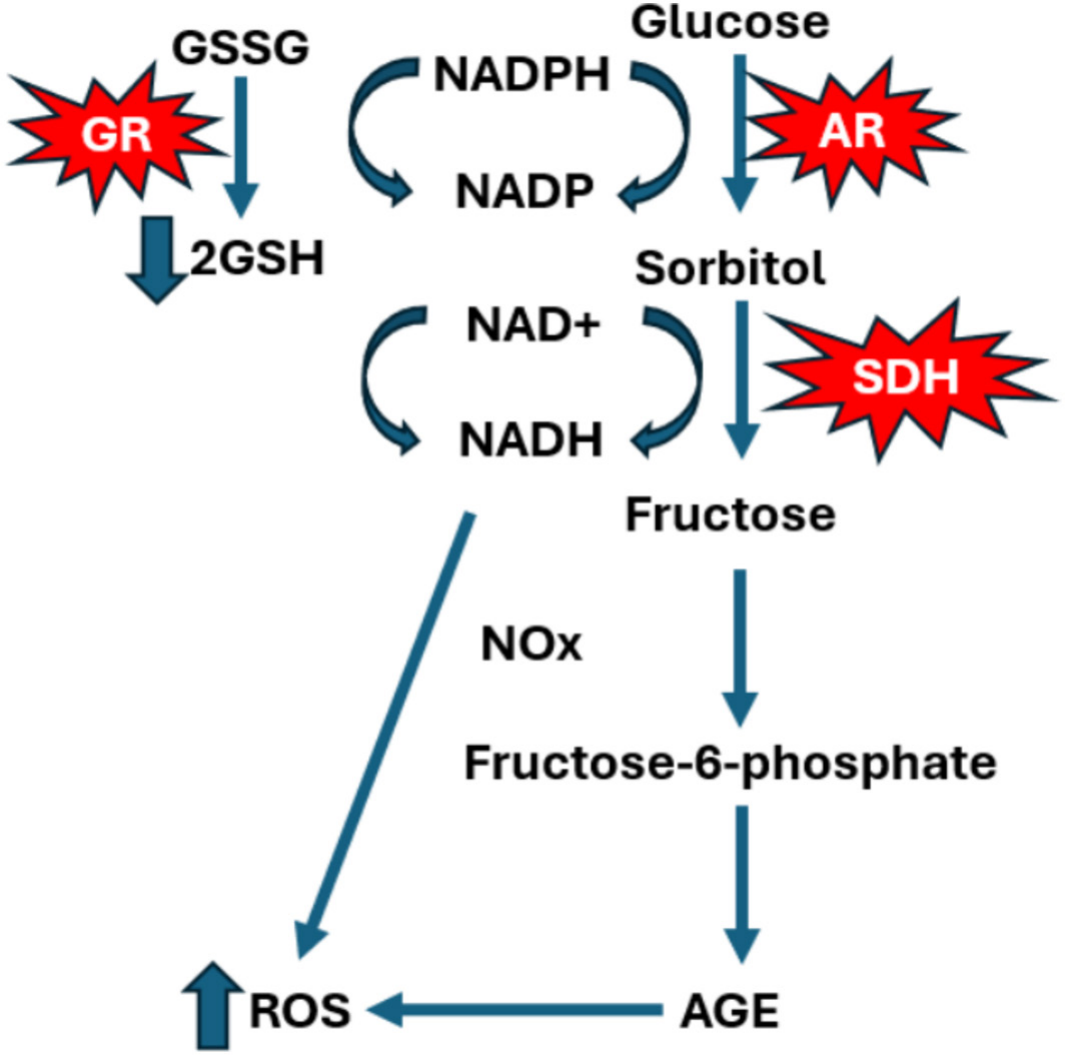
The polyol pathway induces oxidative stress through several mechanisms. AR competes with GR for the cofactor NADPH, reducing its availability for GR and leading to decreased levels of GSH, weakening the cell's antioxidant defenses. SDH activity converts sorbitol to fructose, generating NADH. Elevated NADH activates NADH oxidase (Nox), resulting in the production of ROS. Fructose metabolism produces fructose‐6‐phosphate as an intermediate, promoting AGE formation, collectively contributing to oxidative stress and tissue damage. Aldose reductase (AR), sorbitol dehydrogenase (SDH), nicotinamide adenine dinucleotide (NAD+), glutathione (GSH), glutathione disulfide (GSSG), nicotinamide adenine dinucleotide oxidase (Nox), nicotinamide adenine dinucleotide phosphate (NADPH).

SDH oxidizes sorbitol to fructose, which consumes NAD^+^ and generates NADH. The resulting NADH is a substrate for NADH oxidase, which promotes the production of ROS, directly contributing to oxidative stress [24]. Furthermore, the polyol ‘pathway's product, fructose, and its highly reactive metabolites exacerbate non‐enzymatic glycation processes, leading to the formation of AGEs and further intensifying oxidative damage [25], as shown in Figure 1.

This paper synthesizes current molecular, endocrine, and clinical evidence on how DM‐related oxidative stress impairs male fertility and evaluates potential strategies for mitigating its impact, including antioxidant therapy, lifestyle modification, and optimal glycemic control.

## 2 Oxidative Stress and Sperm Parameters

Several clinical studies have investigated the impact of DM on male reproductive health, with a focus on semen quality and fertility outcomes (Table 1). Ali et al. [26], assessed semen characteristics in a large cohort of 414 diabetic men (100 with insulin‐dependent diabetes mellitus and 314 with non‐insulin‐dependent diabetes mellitus compared to age‐matched non‐diabetic controls). The authors reported that diabetic participants exhibited lower sperm motility and reduced semen volume. Sperm concentration and total count were comparable to controls in men without neuropathy but were elevated in those with neuropathy. Normal sperm morphology was similar between controls and diabetic men without neuropathy but was significantly reduced in diabetic men with neuropathy.

**TABLE 1:** Studies evaluating the impact of DM‐related oxidative stress on conventional semen parameters.

Author	Study design	#Participants	Semen volume (SV)	Sperm concentration	Sperm total count	Sperm motility	Sperm normal morphology	Sperm vitality
Ali et al. [26]	Cross‐sectional (comparative)	414 diabetic men (100 IDDM, 314 NIDDM) + age‐matched non‐diabetic controls	Significantly ↓ in diabetics (≈60% lower)	Significantly ↑ in diabetics with neuropathy (*p* < 0.05)	Significantly ↑ in diabetics with neuropathy (*p* < 0.05)	~30% ↓ in diabetics	No significant difference	Not reported
Agbaje et al. [27]	Case–control (fertility clinic, Ireland)	27 insulin‐dependent diabetic men vs. 29 age‐matched healthy controls	↓Significantly lower in diabetics (*p* < 0.05)	No significant difference	No significant difference	No significant difference	No significant difference	Not assessed/Not reported
Facondo et al. [28]	Systematic review + meta‐analysis	380 T1DM vs. 434 controls	No significant difference	No significant difference	No significant difference	↓ Progressive motility (−33.6%, *p* < 0.001)	↓ Normal morphology (SMD –0.36, *p* < 0.05)	Not reported
Pergialiotis et al. [29]	Meta‐analysis	405 T2DM men vs. 279 non‐diabetic controls	Significantly ↓ in diabetics (*p* < 0.05)	Not reported	No significant difference	No significant difference	No significant difference	Not reported
Rama Raju et al. [30]	Case–control (assisted reproduction clinic, India)	35 T2DM men vs. 123 non‐diabetic controls	No significant difference	Not reported	No significant difference	Significantly ↓ progressive motility (*p* < 0.02)	No significant difference	Not reported
Singh et al. [31]	Case–control (infertility clinic, India)	25 T2DM men vs. 25 non‐diabetic controls	Significantly ↓ in diabetics	Not reported	Significantly ↓ in diabetics	Significantly ↓ in diabetics (52.3% vs. 63.1%)	Not reported	Not reported
La Vignera et al. [32]	Case–control (andrology clinic, Italy)	32 T1DM men vs. 20 fertile controls	No significant difference (*p* = 0.97)	No significant difference (*p* = 0.26)	Not reported	Significantly ↓ in diabetics (*p* < 0.01)	No significant difference (*p* = 0.72)	Not reported
Inih et al. [33]	Cross‐sectional (case–control)	108 T2DM men vs. 56 age‐matched controls	Not reported	Not reported	Significantly ↓ in diabetics (*p* < 0.001)	Significantly ↓ in diabetics (*p* < 0.001)	Significantly ↓ in diabetics (*p* < 0.001)	Not reported
Mallidis et al. [34]	Case–control (university clinic, UK)	13 T1DM men vs. 9 non‐diabetic controls	No significant difference	No significant difference	Not analyzed due to discordant control values	No significant difference	No significant difference	Not reported
Bhattacharya et al. [35]	Case–control (fertility clinic, Kolkata, India)	52 T2DM men vs. 66 fertile non‐diabetic controls	Significantly ↓ in diabetics (*p* = 0.004)	No significant difference (*p* = 0.06)	Significantly ↓ in diabetics (*p* = 0.01)	Significantly ↓ in diabetics (*p* < 0.0001)	Significantly ↓ in diabetics (*p* = 0.02)	Not reported
Lu et al. [36]	Case–control (fertility clinic, Wenzhou, China)	30 diabetic men vs. 30 healthy controls	Significantly ↓ in diabetics (*p* < 0.05)	Not reported	Significantly ↓ in diabetics (*p* < 0.05)	Significantly ↓ in diabetics (*p* < 0.05)	Significantly ↓ in diabetics (*p* < 0.05)	Significantly ↓ in diabetics (*p* < 0.05)

*Note:* ↓ = decrease and ↑ = increase sperm parameters.

Agbaje et al. [27], in a study of 27 insulin‐dependent diabetic men and 29 age‐matched controls, reported significantly lower semen volume in the diabetic group, while sperm motility, morphology, and concentration were comparable between groups.

Facondo et al. [28], in a study involving 380 men with T1DM and 434 non‐diabetic controls, reported that diabetic participants exhibited significantly lower progressive sperm motility and a reduced proportion of morphologically normal spermatozoa, while semen volume and sperm concentration remained comparable between groups. La Vignera et al. [32], compared 32 men with T1DM to 20 fertile controls and found that progressive sperm motility was lower in the diabetic group, whereas sperm concentration, semen volume, and morphology did not differ between groups.

Mallidis et al. [34], in a small cohort of 13 men with T1DM and 9 non‐diabetic controls, assessed the presence of AGEs in the male reproductive tract. While most semen parameters did not differ significantly between groups, total sperm count was reported as higher in T1DM men. Notably, levels of carboxymethyl‐lysine, a major AGE, were significantly lower in the spermatozoa of T1DM participants.

Among studies focused on T2DM, Singh et al. [31], compared 25 controls to 25 men with T2DM and observed reductions in sperm motility, semen volume, and total sperm count in the diabetic group. Bhattacharya et al. [35], analyzing 66 non‐diabetic fertile controls and 52 men with T2DM, similarly reported reduced semen volume, sperm concentration, motility, and morphology in the diabetic group. Inih et al. [33], compared 108 men with T2DM to 56 controls and found no differences in semen volume or sperm vitality; however, total sperm count, motility, and morphology were reduced in the diabetic group.

Lu et al. [36], compared 30 healthy controls to 30 men with diabetes and reported that the diabetic group had significantly lower semen volume, total sperm count, normal morphology, sperm vitality, and motility compared to non‐diabetic men.

Rama Raju et al. [30], in a comparative analysis involving 35 men with type 2 diabetes mellitus (T2DM) and 123 non‐diabetic controls, a significant reduction in sperm motility among diabetic men was observed, while no significant differences were observed between the groups in the other semen parameters assessed.

Pergialiotis et al. [29], comparing 405 men with T2DM and 279 controls, reported lower semen volume in the diabetic group, but no differences in sperm concentration, total count, motility, or morphology.

## 3 Effects of Oxidative Stress on Male Sexual Hormones

Male reproductive function is tightly controlled by the HPG axis, which regulates testosterone production and the secretion of gonadotropins, such as follicle‐stimulating hormone (FSH) and luteinizing hormone (LH) [37]. Conditions such as diabetes, obesity, and hypertension can disrupt this axis, resulting in altered hormone levels and impaired fertility (Table 2) [45]. Characterizing these hormonal changes is essential for understanding the mechanisms underlying male subfertility and guiding potential therapeutic interventions.

**TABLE 2:** Studies evaluating serum FSH, LH, and TT levels in men with diabetes mellitus or related metabolic conditions compared to control groups.

Author	Type of study	#Subjects	(FSH)	(LH)	(TT)
Onwubuya et al. [38]	Prospective case–control	90 hypertensive vs. 60 controls	Higher in hypertensive	Higher in hypertensive	Lower in hypertensive
Rehman et al. [39]	Cross‐sectional case–control	241 infertile vs. 135 fertile controls	Higher in infertile	Higher in infertile	Higher in fertile
AbbasiHormozi et al. [40]	Cross‐sectional	40 control vs. 40 obese vs. 35 lean diabetic vs. 35 obese diabetic	No significant difference	No significant difference	Controls: higher; all affected groups lower (*p* < 0.05); lowest in obese diabetic
Hashim et al. [41]	Prospective	158 DM (T1DM & T2DM) vs. 79 controls	T2DM: lower (*p* < 0.05); T1DM: higher (*p* < 0.05)	T2DM: lower (*p* < 0.05); T1DM: higher (*p* < 0.05)	Lower in both T1DM & T2DM
Grossmann et al. [42]	Cross‐sectional	580 T2DM vs. 69 T1DM	Not reported	Not reported	Low in 43% of T2DM vs. 7% T1DM
Uzor et al. [43]	Cross‐sectional	30 diabetic vs. 30 controls	Not reported	Not reported	Lower in diabetics
Yeste et al. [44]	Cross‐sectional	38 T1DM vs. 55 T2DM vs. 100 controls	No difference	Higher in T2DM	Lower in T2DM

Onwubuya et al. [38], in a prospective case–control study, assessed the effect of oxidative stress on reproductive hormones in 90 newly diagnosed hypertensive males and 60 normotensive controls aged 30–65 years. Serum levels of FSH, LH, and TT were evaluated. The authors reported that hypertensive subjects demonstrated significantly elevated FSH (11.94 ± 4.14 vs. 7.16 ± 3.40 mIU/mL, *p* < 0.001) and LH (8.46 ± 2.54 vs. 3.31 ± 1.74 mIU/mL, *p* < 0.001) compared to controls, while TT levels were significantly reduced (3.19 ± 2.63 vs. 7.32 ± 1.54 ng/mL, *p* < 0.001).

Rehman et al. [39], conducted a cross‐sectional case–control study involving 376 men aged 25–55 years, comprising 241 infertile males and 135 fertile controls. The authors reported an increase in serum FSH and LH hormones in cases compared to the controls (*p* = 0.05), whereas TT levels were higher in the controls (*p* < 0.001). The findings further indicated that oxidative stress and reduced antioxidant enzyme activity contributed to impaired testicular function, thus playing a critical role in male infertility.

Abbasi Hormozi et al. [40], evaluated the impact of obesity and diabetes on reproductive hormones in subfertile men across four groups: control (*n* = 40), obese (*n* = 40), lean diabetic (*n* = 35), and obese diabetic (*n* = 35). Serum TT and sex hormone‐binding globulin (SHBG) concentrations were significantly reduced in all three affected groups compared with controls (*p* < 0.05). The lowest TT and SHBG levels were observed in obese diabetic men, with no change in LH or FSH levels.

In a prospective study by Hashim et al. [41], involving 158 male individuals with either T1DM or T2DM and 79 age‐matched healthy controls, serum reproductive hormones were compared to evaluate HPG axis function. Men with T2DM exhibited significantly lower TT, LH, and FSH levels (*p* < 0.05) compared to controls, indicating a secondary hypogonadism pattern with hyperprolactinemia. In contrast, men with T1DM demonstrated significantly lower TT levels (*p* < 0.05) but significantly higher LH and FSH levels (*p* < 0.05) compared to both T2DM patients and controls, consistent with primary testicular failure.

Grossmann et al. [42], conducted a cross‐sectional survey involving 580 men with T2DM and 69 men with T1DM to assess the prevalence of testosterone deficiency and its association with insulin resistance. Reduced TT was found in 43% of men with T2DM compared to 7% of men with T1DM, while reduced calculated free testosterone (cFT) was present in 57% of T2DM and 20.3% of T1DM men (age‐ and BMI‐adjusted OR = 1.4; 95% CI: 0.7–2.9). Low TT was independently associated with insulin resistance in both types of diabetes. In a subgroup of 262 men with T2DM reassessed after a median of 6 months, changes in TT levels were inversely related to changes in insulin resistance.

Uzor et al. [43], conducted a cross‐sectional study involving 30 diabetic male patients and 30 age‐matched non‐diabetic healthy controls to assess the effect of diabetes on testosterone. Diabetic participants had significantly lower serum testosterone levels compared to controls (*p* < 0.05), with no changes in LH and FSH.

Yeste et al. [44], conducted a cross‐sectional study including 38 men with T1DM, 55 men with T2DM, and 100 healthy fertile controls. TT was lower in T2DM (4.1 ± 0.5 ng/mL) compared to controls (5.1 ± 0.1 ng/mL) and T1DM (6.1 ± 1.1 ng/mL), although the difference was statistically significant. LH was significantly higher in T2DM (5.6 ± 0.1 UI/L) compared to T1DM (2.1 ± 0.1 UI/L) and controls (2.4 ± 0.1 UI/L) (*p* < 0.05). FSH showed no significant difference among groups (controls: 2.9 ± 0.9 UI/L; DM1: 2.3 ± 0.3 UI/L; DM2: 2.5 ± 0.1 UI/L). The authors suggested that T2DM is associated with lower testosterone and elevated LH, consistent with primary hypogonadism, whereas T1DM generally preserved testosterone but showed other sperm quality impairments.

## 4 Effect of Oxidative Stress on Rates of Children (Natural Fertility) and Assisted Reproduction Technologies (ART) Outcomes

Oxidative stress is increasingly recognized as a critical factor in the decline of natural male fertility, with chronic metabolic conditions such as diabetes mellitus playing a central role [46]. Men with DM, particularly T2DM, exhibit significantly lower rates of natural conception compared to healthy counterparts [47]. This reduction in fertility is multifactorial but strongly linked to the persistent elevation of ROS driven by hyperglycemia and insulin resistance [48]. Additionally, chronic hyperglycemia disrupts the HPG axis, often suppressing LH and FSH and subsequently impairing Leydig cell function and testosterone synthesis [49].

Cross‐sectional observational study conducted by Wiebe et al. [50], investigated the effect of T1DM on natural fertility, using data from the Type 1 Diabetes Genetics Consortium. The study included 3010 adults with T1DM and 801 unaffected siblings, and fertility was assessed based on the number of offspring. The authors reported a significant reduction in fertility among individuals with T1DM compared to their unaffected siblings (*p* < 0.001), with a pronounced effect in women (fertility ratio: 0.72; 95% CI: 0.6–0.85) compared to men (0.91; 95% CI: 0.64–0.98). Further analysis revealed that individuals with adult‐onset diabetes had more children than those with childhood‐onset (*p* < 0.001), while disease duration had no significant effect.

In a large population‐based retrospective cohort study conducted in Finland, Sjöberg et al. [51], investigated the effect of childhood‐onset T1DM on fertility. The study included 2307 women and 2819 men diagnosed with T1DM before age 18 between 1965 and 1979, along with two matched non‐diabetic controls for each case. The authors reported that both diabetic women and men had significantly fewer live births compared to controls. The hazard ratio (HR) for having a first child was 0.66 (95% CI: 0.62–0.71; *p* < 0.001) in women and 0.77 (95% CI: 0.72–0.83; *p* < 0.001) in men. Later onset of diabetes was associated with increased likelihood of having children, a higher rate of first childbirth in men (*p* = 0.04) and second childbirth in women (*p* = 0.002).

In a cross‐sectional study, Holstein et al. [52], evaluated fertility patterns and offspring sex ratio in 697 German adults with T1DM (364 women, 333 men) compared to national population data. Women with T1DM (age 18–49 years) had a mean fertility rate of 0.88 children compared to 1.36 in the general female population, while men had an even lower rate of 0.65. Childlessness was more common among men (51.1%) than women (35.7%), and substantially higher in both sexes compared to background rates. Additionally, the sex ratio of offspring (female: male) did not significantly differ from the expected 1:1 in either sex, even when only considering children conceived after diabetes onset.

Eisenberg et al. [53], conducted a prospective cohort study within the Longitudinal Investigation of Fertility and the Environment (LIFE) Study, involving 501 couples from Michigan and Texas planning pregnancy. The authors reported that diabetes in male partners was associated with a significant 65% reduction in fecundability (FOR 0.35, 95% CI: 0.14–0.86), persisting in adjusted models (FOR 0.35, 95% CI: 0.13–0.88), while female partners with diabetes showed a similar but non‐significant reduction (FOR 0.26, 95% CI: 0.03–1.98), likely due to lower prevalence.

Mulholland et al. [54], conducted a retrospective chart review of 3000 couples attending a fertility clinic to examine the impact of male DM on ART outcomes. Eighty couples (2.7%) had a male partner with DM, of which 18 proceeded to ART (5 IVF, 12 ICSI, 1 both). Two men (11%) presented with retrograde ejaculation, and two were azoospermic. Fertilization rates were comparable to non‐diabetic patients (IVF: 68% vs. 70%; ICSI: 62% vs. 71%), and no differences in embryo quality were reported. Nonetheless, the combined clinical pregnancy rate per transfer for fresh IVF and ICSI cycles was significantly lower in the DM group (3.3%) compared with the clinic average (27.9%). By contrast, the pregnancy rate for frozen embryo transfer (FET) was 29%, similar to the expected 21.3%.

## 5 Oxidative Stress in Fertile and Infertile Men

Fertile men are not exempt from oxidative stress, but maintain adequate antioxidant defenses, as a physiological level of ROS is necessary for normal sperm functions such as capacitation, the acrosome reaction, and sperm‐oocyte interaction [55]. In fertile individuals, oxidative stress biomarkers such as 8‐hydroxy‐2′‐deoxyguanosine (8‐OHdG) and malondialdehyde (MDA) are generally found at lower concentrations compared to infertile men [56]. Several studies have investigated the oxidative profile in both fertile and infertile men, highlighting consistent differences in redox status (Table 3) [39, 56, 57, 58, 59, 60, 61, 62, 63, 64, 65, 66]. Recent evidence suggests that infertile men typically exhibit significantly elevated levels of oxidative stress markers, alongside reduced activity in antioxidant enzymes such as catalase, superoxide dismutase (SOD), and lower total antioxidant capacity (TAC) [66].

**TABLE 3:** Comparison of oxidative stress biomarkers between fertile and infertile men across selected clinical studies, highlighting differences in antioxidant enzyme activities and oxidative damage markers.

Author	Type of study	Subjects	Outcome in fertile men	Outcome in infertile men
Pasqualotto et al. [57]	Prospective	17 fertile men (control) vs. 21 infertile varicocele men	Low ROS, high TAC, optimal semen quality score	High ROS, lower TAC, and low semen quality
Atig et al. [58]	Cross‐sectional	40 fertile men (control) vs. 80 infertile men	High SOD, GPx, GSH, and Zinc Low MDA content	Low antioxidants High MDA content
Mahanta et al. [59]	Case–control	20 fertile men (control) vs. 50 infertile men	Low Lipid peroxide (LPO) and Protein peroxide (PPO) High SOD and GPx in blood and semen	High LPO and PPO content Low SOD and GPx in blood and semen
Aktan et al. [60]	Prospective	14 fertile men (control) vs. 28 idiopathic males	Low sperm DNA fragmentation Low ROS, MDA content, protein carbonyl (PC), and nitrotyrosine (NT)	High sperm DNA fragmentation and ROS Elevated seminal MDA, PC, and NT
Wdowiak et al. [61]	Cross‐control	85 fertile men (control) vs. 133 infertile men	High SOD activity Low sperm DNA fragmentation over 12 h post‐donation	Low SOD activity High sperm DNA fragmentation, especially between 6 and 12 h post‐donation
Dorostghoal et al. [62]	Cross‐sectional	105 fertile men (control) vs. 112 infertile men	Low MDA content, high SOD, and GPx Low sperm DNA fragmentation	High MDA content, Low SOD, and GPx High sperm DNA fragmentation
Palani et al. [63]	Case–control	21 fertile men (control) vs. 46 infertile men (normozoospermia)	Normal levels of TAC, glutathione, MDA, uric acid, and albumin	Significantly reduced TAC (Serum and seminal; *p* < 0.0001); no significant difference in other markers
Rehman et al. [39]	Cross‐sectional (case–control)	135 fertile men (control) vs. 241 infertile men	Low cortisol and adrenaline, High antioxidants (GSH, GPx, and SOD)	Significantly higher cortisol and adrenaline (*p* < 0.05) Low antioxidants
Aljaser et al. [64]	Cross‐sectional (comparative study)	40 fertile men (control) vs. 30 infertile men	Low ROS and MDA content, High TAC, normal trace elements (Zn, Mg)	High ROS and MDA content Low TAC and trace elements (Zn, Mg)
Yusuf et al. [56]	Cross‐sectional (Observational)	30 fertile men (control) vs. 70 infertile men	Low 8‐OHdG and MDA content High TAC and SOD	High 8‐OHdG and MDA content Low TAC and SOD levels
Ahmed et al. [65]	Cross‐sectional (Comparative)	60 fertile men (control) vs. 60 infertile men	Decreased MDA content Increased SOD and glutathione peroxidase	Increased MDA content Decreased SOD and glutathione peroxidase

In a prospective study, Pasqualotto et al. [57], evaluated semen quality and oxidative stress markers among 21 infertile men with varicocele, 15 fertile men with varicocele, and 17 fertile controls without varicocele. The study revealed that the varicocele group demonstrated a lower semen quality score compared to the fertile control group. No significant difference was observed between infertile and fertile varicocele patients. Furthermore, infertile men with varicocele exhibited elevated ROS levels and reduced TAC compared to fertile controls.

Atig et al. [58], in a cross‐sectional study, evaluated antioxidant status and lipid peroxidation in the seminal plasma of 120 Tunisian infertile men undergoing IVF, grouped into normozoospermic (*n* = 40), asthenozoospermic (*n* = 45), and oligoasthenoteratozoospermic (OAT; *n* = 35). The control group showed significantly higher superoxide dismutase (SOD) (*p* = 0.008; *p* = 0.003) and glutathione peroxidase (GPX) (*p* = 0.02; *p* < 0.001) activities compared to the asthenozoospermic and OAT groups, respectively. Zinc and reduced glutathione (GSHr) levels were also elevated in controls (*p* < 0.001 and *p* = 0.008), while malondialdehyde (MDA) levels were significantly increased in both infertile groups (*p* < 0.001). Furthermore, SOD and GPX activities positively correlated with sperm motility and count, whereas MDA was negatively correlated with motility (r = −0.47, *p* < 0.001).

Mahanta et al. [59], conducted a comparative case–control study to investigate the role of oxidative stress in male infertility among men from North‐East India. A total of 50 infertile men were compared with 20 normozoospermic fertile controls. The results showed significantly elevated levels of lipid peroxide (LPO) and protein peroxide (PPO) in both blood and semen samples of infertile males. Blood LPO and PPO levels were 60.84 ± 3.55 nmol/mL and 72.84 ± 3.66 nmol/mL, respectively, compared to 40.20 ± 4.33 nmol/mL and 59.93 ± 5.24 nmol/mL in controls (*p* < 0.001). Superoxide dismutase (SOD) and glutathione peroxidase (GPX) were significantly reduced in infertile men in both blood (SOD: 3.40 ± 1.06 U/mL; GPX: 0.16 ± 0.01 U/mL) and semen (SOD: 2.42 ± 1.32 U/mL; GPX: 0.24 ± 0.015 U/mL) compared to fertile controls (blood SOD: 4.85 ± 0.78 U/mL; GPX: 0.36 ± 0.05 U/mL; semen SOD: 4.24 ± 0.89 U/mL; GPX: 0.65 ± 0.03 U/mL; *p* < 0.001).

Aktan et al. [60], conducted a prospective case–control study to investigate the role of OS in idiopathic male infertility among Turkish men. Semen samples from 28 men with idiopathic infertility were compared to 14 fertile controls. The results showed significantly higher levels of sperm DNA fragmentation (*p* < 0.001) and intracellular ROS (*p* < 0.001) in the infertile cohort. Furthermore, OS markers in seminal plasma, malondialdehyde (*p* < 0.001), protein carbonyls (*p* = 0.003), and nitrotyrosine (*p* = 0.006) were also elevated in the infertile group. Positive correlations were found between DNA fragmentation and MDA, PC, and NT levels, as well as between ROS content and MDA.

Wdowiak et al. [61], in a comparative cross‐sectional study involving 85 fertile men and 133 infertile patients (aged 25–35), demonstrated that the seminal superoxide dismutase (SOD) activity was significantly lower in infertile men (36.05 U/mL) compared to fertile men (42.93 U/mL) (*Z* = −12, *p* < 0.001). In contrast, catalase activity showed no significant difference between the groups. Sperm DNA fragmentation index (DFI) was significantly higher in infertile men at 0 h (*p* < 0.001), 3 h (*p* = 0.02), and 12 h (*p* < 0.001), but not at 6 h (*p* = 0.084). Among infertile men, a negative correlation existed between SOD activity and DFI at 12 h (*r* = −0.202, *p* = 0.020), while catalase activity positively correlated with initial DFI (*r* = 0.210, *p* = 0.016).

Dorostghoal et al. [62], in a comparative cross‐sectional study involving 105 fertile and 112 infertile men in southwest Iran, reported significantly elevated levels of MDA in the seminal plasma of infertile men (*p* < 0.001). SOD and GPx were significantly reduced in infertile men (*p* < 0.001). Sperm DNA fragmentation was also higher in infertile subjects (*p* < 0.001) and positively correlated with MDA while negatively correlated with SOD and GPx activities. The authors identified cut‐off values of 4.2 nmol/mL for MDA, 4.89 U/mL for SOD, and 329.6 mU/mL for GPx to distinguish infertile from fertile men.

Palani et al. [63], in a case‐control study, assessed oxidative stress markers and antioxidant activity in 46 normozoospermic infertile men and 21 fertile controls. All infertile participants had normal semen parameters but experienced infertility for more than 12 months, while fertile controls had fathered a child within the last year. The authors reported significantly higher ROS levels (*p* < 0.001) and increased sperm DNA fragmentation (SDF) (*p* < 0.001) in the infertile group. In contrast, levels of TAC and catalase activity were significantly reduced in infertile men compared to fertile controls (*p* < 0.001 for both). Furthermore, ROS levels showed a positive correlation with SDF (*r* = 0.615, *p* < 0.001) and a negative correlation with both TAC (*r* = −0.531, *p* < 0.001) and catalase activity (*r* = −0.537, *p* < 0.001).

Rehman et al. [39], conducted a cross‐sectional case–control study involving 376 men aged 25–55 years, comprising 241 infertile males and 135 fertile controls. The infertile group demonstrated significantly elevated median levels of cortisol, adrenaline, SOD, and GPx compared to the fertile controls (*p* < 0.05). The authors reported that for every 7‐unit increase in cortisol, the likelihood of infertility increased by 3% (*p* = 0.001).

Aljaser et al. [64], in a cross‐sectional study, evaluated a total of 70 semen samples, including 40 normozoospermic fertile controls and 30 infertile asthenozoospermic men. The infertile group exhibited significantly higher ROS (*p* < 0.001) and MDA levels (*p* < 0.01), alongside significantly lower TAC (*p* < 0.01), Zn (*p* < 0.01), and Mg (*p* < 0.01) compared to fertile controls. While Se and Cu levels were not significantly different between groups, Se showed a slight increase in fertile males. The authors reported significant positive correlations between Mg, Se, and Zn levels and sperm morphology and motility (Zn and motility: *r* = 0.381, *p* < 0.01; Se and morphology: *r* = 0.36, *p* < 0.01), while ROS and MDA levels correlated negatively with motility and morphology (ROS and morphology: *r* = −0.51, *p* < 0.001).

Yusuf et al. [56], conducted a cross‐sectional study involving 70 infertile males in Benin City, Nigeria. Participants were classified as normozoospermic (*n* = 20), oligozoospermic (*n* = 30), or azoospermic (*n* = 20), with a control group of fertile men (*n* = 30). The authors reported that infertile subjects had significantly higher levels of 8‐OHdG and MDA (*p* = 0.01), with the highest values in azoospermic men. TAS and SOD levels were significantly lower in infertile men (*p* = 0.01), decreasing progressively from normozoospermia to azoospermia. Moreover, 8‐OHdG negatively correlated with sperm count (*r* = −0.360, *p* = 0.01), motility (*r* = −0.388, *p* = 0.04), and morphology (*r* = −0.327, *p* = 0.02), indicating a strong association between oxidative DNA damage and impaired semen quality.

Ahmed et al. [65], conducted a cross‐sectional comparative study involving 120 participants (60 infertile men and 60 fertile controls) in Kano, Northwestern Nigeria. There was a significant increase in MDA levels among the infertile group (1.71 ± 0.39 nmol/mL) compared to controls (0.95 ± 0.14 nmol/mL; *p* < 0.0001). The SOD (1.67 ± 0.40 IU/L vs. 1.84 ± 0.52 IU/L; *p* = 0.0389) and GPx (54.26 ± 13.79 IU/L vs. 72.03 ± 15.29 IU/L; *p* < 0.0001) levels were significantly decreased in the infertile group. Interestingly, 80% of the infertile subjects presented with primary infertility, and 70% were diagnosed with oligozoospermia, while 26.7% had azoospermia. The mean age did not differ significantly between groups (infertile: 36.68 ± 5.31 years; control: 36.47 ± 6.64 years; *p* = 0.8439), though body mass index (BMI) was significantly higher in infertile men (*p* = 0.0287).

## 6 Impact of Obesity on Sperm Parameters and Male Fertility

Metabolic syndrome (MetS) and obesity are increasingly recognized as significant contributors to male infertility. MetS comprises a cluster of interrelated metabolic abnormalities, including hypertension, insulin resistance, central obesity, and dyslipidemia that collectively impair endocrine and reproductive function [67]. Obesity, both as an independent condition and a central component of MetS, has been shown to affect spermatogenesis and overall semen quality (Table 4) through multiple mechanisms [74].

**TABLE 4:** Studies evaluating the relationship between BMI and male reproductive function, highlighting effects on semen quality, hormonal profile, and fertility outcomes.

Author	Study design and sample	Methodology	Key findings	Conclusions
Huetos et al. [68]	Meta‐analysis (28 studies)	Random‐effects meta‐analysis of BMI and sperm parameters	Overweight and obese men had lower semen volume, sperm count, concentration, motility, vitality, and morphology	Male adiposity is inversely associated with sperm quality; weight management is essential
Li et al. [69]	Meta‐analysis (50 studies, ~71 000 men)	Random‐effects model; pooled semen parameters by BMI	Obese men had significantly lower sperm volume, count, motility, and morphology; severity increased with obesity class	Adiposity negatively affects all sperm parameters; supports weight loss interventions
Zhu et al. [70]	Cross‐sectional (54 subfertile men)	Semen analysis, DNA fragmentation assay, and apoptosis markers via microarray	Obese men had higher DNA fragmentation, lower motility, and upregulated apoptotic proteins (Fas/FasL, p53, caspase‐3)	Obesity promotes sperm apoptosis and DNA damage through oxidative stress and apoptotic pathways
Amoah et al. [71]	Cross‐sectional (212 men, IVF clinic – Ghana)	WHO semen analysis, hormone assays; and BMI classification	Elevated BMI is linked to lower sperm morphology and testosterone, but higher semen volume and viability	BMI impacts both hormonal and structural sperm parameters, a modifiable infertility factor
Service et al. [72]	Systematic review (112 studies)	Qualitative synthesis of human and animal studies	Obesity and metabolic syndrome impair sperm quality, DNA integrity, and hormone levels	Lifestyle modifications (e.g., weight loss, exercise) may reverse damage
Sermondade et al. [73]	Meta‐analysis (21 studies; 13 077 men)	Odds ratios for oligozoospermia/azoospermia across BMI	J‐shaped relationship: OR = 2.04 for morbid obesity; increased risk also in underweight	Both low and high BMI increase risk of abnormal sperm count; optimal BMI is essential

Huetos et al. [68], conducted a systematic review and meta‐analysis to quantitatively evaluate the relationship between male adiposity and sperm quality. The authors systematically screened 169 studies and included 28 quantitative studies that met their inclusion criteria. These studies evaluated the relationship between body mass index (BMI) categories normal weight, overweight, and obese and various conventional sperm parameters. The meta‐analysis revealed that both overweight and obese men exhibited significantly lower semen volume, sperm concentration, total sperm count, vitality, motility, and the percentage of sperm with normal morphology when compared to men with normal BMI. Furthermore, the authors highlighted potential mechanisms linking excess adiposity to impaired spermatogenesis, as well as hormonal imbalances, oxidative stress, increased scrotal temperature, and inflammation.

In a large‐scale meta‐analysis, Li et al. [69] synthesized findings from 50 studies, including over 71 000 participants, to assess the association between semen quality and BMI. The authors reported that obese men had significantly reduced semen volume, total sperm count, progressive motility, and morphology compared to men of normal weight. The severity of impairment increased with higher classes of obesity, highlighting a dose–response relationship.

A cross‐sectional study by Zhu et al. [70], investigated the association between body mass index (BMI) and sperm apoptosis in 54 subfertile men, stratified into normal weight, overweight, and obese groups. While no significant differences were found in semen volume, sperm concentration, or morphology across groups, obese men showed increased sperm DNA fragmentation and apoptosis rates, as well as reduced progressive motility. Using antibody microarray analysis, the study further revealed that apoptosis‐related proteins such as Fas/FasL, Bcl‐2/Bax, caspase‐3, caspase‐8, p53, and p21 were upregulated in overweight and obese individuals.

In a cross‐sectional study conducted in Ghana by Amoah et al. [71], the association between BMI and male reproductive parameters was evaluated among 212 men attending an IVF clinic. The results revealed that elevated BMI was significantly associated with lower testosterone levels and reduced sperm morphology, suggesting impaired spermatogenesis and hormonal imbalance in overweight and obese men. Interestingly, higher BMI also showed a positive correlation with semen volume and sperm viability, indicating possible compensatory responses or confounding lifestyle influences.

Additionally, a systematic review by Service et al. [72], evaluated 112 studies examining the effects of obesity, diabetes, and metabolic syndrome on male fertility. The authors conducted a qualitative synthesis of findings across human and animal models, emphasizing the consistency of negative associations between BMI and semen quality, sperm DNA integrity, and endocrine function. The review also evaluated intervention studies, concluding that lifestyle modifications such as exercise, caloric restriction, and bariatric surgery may improve semen parameters and hormonal balance.

Sermondade et al. [73], conducted a systematic review and collaborative meta‐analysis focusing on the relationship between BMI and risks of oligozoospermia and azoospermia. The study included 21 observational studies, encompassing a total of 13 077 men from both the general population and fertility clinics. The analysis revealed a J‐shaped relationship between BMI and the risk of oligozoospermia or azoospermia. Compared to men with normal BMI, the odds ratios (95% confidence intervals) for oligozoospermia or azoospermia were: underweight, 1.15 (0.93–1.43); overweight, 1.11 (1.01–1.21); obese, 1.28 (1.06–1.55); and morbidly obese, 2.04 (1.59–2.62). The authors argued that the J‐shaped trend reflects both nutritional deficiency and adiposity‐related pathophysiology affecting testicular function.

## 7 Sources of ROS in Seminal Plasma

ROS are naturally produced throughout metabolism and play crucial roles in homeostasis and cell signaling [75]. Evidence suggests certain physiological levels of ROS actively participate in the process of sperm‐oocyte fusion, hyperactivation, capacitation, acrosome reaction, mitochondrial stability, and spermatozoa maturation [76], however, excessive formation of ROS, on the other hand, surpasses the capacity of antioxidants in the seminal plasma to neutralize them, potentially leading to adverse effects that may damage testicular tissue and result in male infertility [77]. The formation of ROS may either be exogenous or endogenous, and the body's antioxidant defense system works to counteract the potentially damaging effects of pro‐oxidant molecules [78].

### 7.1 Exogenous Sources of ROS

Exogenous factors such as exposure to industrial heavy metals, alcohol consumption, smoking, radiation, diet, sedentary lifestyle, and temperature have been associated with male infertility and increased ROS [79]. Cigarette smoking introduces a plethora of toxins, carcinogens, and mutagenic substances into the body [80], each capable of accelerating oxidative stress.

The primary mechanism by which smoking affects male fertility is through the direct induction of ROS formation. Tobacco smoke contains chemicals that are potent inducers of oxidative stress, leading to a decrease in sperm concentration, morphology, and motility [81]. Moreover, smoking triggers a systemic inflammatory response, evidenced by the recruitment of leukocytes into the seminal plasma [82, 83]. These immune cells, while defending against the perceived threat, paradoxically contribute to the male infertility narrative by generating excessive ROS. This oxidative onslaught results in significant sperm DNA fragmentation and axonemal damage [83].

Excessive alcohol consumption has been unequivocally linked to a spectrum of detrimental effects on male reproductive health [84]. The metabolism of ethanol results in the production of acetaldehyde, a compound notorious for its reactive properties, which can interact with both lipids and proteins, thereby catalyzing the generation of ROS and thus causing lipid peroxidation [85].

This interaction not only disrupts cellular integrity but also sets off a cascade of oxidative stress within the seminal environment. Concurrently, the production of NADH during this metabolic pathway intensifies mitochondrial respiratory activity, leading to an overproduction of ROS [86].

Exposure to industrial heavy metals such as lead, cadmium, and others serves as an environmental hazard to male fertility by facilitating the generation of ROS [87]. These metals can catalyze the Fenton reaction [86], where they interact with hydrogen peroxide to generate reactive hydroxyl radicals, one of the most reactive forms of ROS, leading to oxidative damage of sperm cells and their DNA damage [78].

Radiation, on the other hand, poses a different but equally sinister threat. Even at low levels, radiation can disrupt cellular homeostasis, particularly mitochondrial function, leading to an increase in OS [88]. Low‐level radiation therapy might impair NADH oxidase activity, which is crucial for ROS neutralization, thereby indirectly promoting sperm death through overwhelming oxidative stress [89].

The anatomical position of the testicles within the scrotum optimizes the thermal environment necessary for spermatogenesis, which is optimally facilitated at a temperature of at least 2°C below that of the core body temperature [90]. Prolonged exposure to heat, whether from frequent use of saunas, hot tubs, or even tight clothing that raises scrotal temperature, can decrease sperm count and motility, in part due to the generation of ROS [91].

Instead of merely serving as an inert energy storage compartment, adipose tissue acts as a dynamic endocrine organ by synthesizing leptin. This adipokine is instrumental in regulating energy balance and precipitating the secretion of proinflammatory cytokines and ROS [92]. Obesity correlates with augmented levels of leptin and ROS, which have notable implications for fertility [93]. Furthermore, the physical accumulation of fat in the groin area can elevate local testicular temperature, mirroring the effects of external heat stress, a condition that has been associated with OS in mouse models [94].

### 7.2 Endogenous Sources of ROS

Varicocele is a medical condition characterized by abnormal dilation of the venous pampiniform plexus around the spermatic cord. It accounts for approximately 40% of infertility cases in male patients, with a general incidence ranging from 4.4% to 22.6% in the adult male population [95]. Meucci et al. [96], reported that OS in men affected by varicocele may result from increased levels of ROS and reduced total antioxidant capacity, leading to impaired cell membrane structure and sperm DNA integrity [97]. Varicocele patients also exhibit a high proportion of spermatozoa with abnormal chromatin structure [98]. Additionally, Ha et al. [99], demonstrated that ROS can induce significant damage to the blood‐testis barrier.

During spermatogenesis, sperm cells undergo a series of biochemical and morphological changes to achieve maturity. However, the process is not without its oxidative challenges. Spermatozoa themselves contribute significantly to the production of ROS [100]. The mitochondria of spermatozoa, localized primarily in the midpiece, are pivotal in this context. During oxidative phosphorylation, electrons may escape from the electron transport chain at complexes I and III, resulting in the generation of ROS, such as superoxide (O_2_^−^), as metabolic byproducts [101, 102]. This superoxide can be further converted to hydrogen peroxide (H_2_O_2_) through the action of superoxide dismutase, exacerbating oxidative stress if not balanced by antioxidants [103].

Leucocytes, including polymorphonuclear cells (PMNs) such as neutrophils and macrophages, are abundant in seminal plasma and represent a primary endogenous source of ROS. These immune cells are recruited to the male reproductive tract, particularly in response to infection or inflammation [16]. The seminal fluid's inflammatory milieu, often observed in conditions like varicocele, encourages leucocyte infiltration, where they produce large amounts of ROS through mechanisms like the respiratory burst, involving NADPH oxidase [104]. This enzymatic pathway is crucial for pathogen defense but can inadvertently damage spermatozoa, leading to compromised fertility due to oxidative damage to sperm DNA, membranes, and motility [105].

The seminal plasma contains various enzymatic systems that can generate ROS. Enzymes like xanthine oxidase (XO) and lipoxygenases are found in seminal secretions [106]. XO contributes to ROS production through purine metabolism, converting hypoxanthine to xanthine and then to uric acid, with superoxide and hydrogen peroxide as byproducts [107]. Lipoxygenases metabolize polyunsaturated fatty acids into lipid hydroperoxides, which are precursors to ROS, contributing to OS in the seminal environment [108].

Peroxisomes are well‐characterized organelles with critical roles in cellular lipid metabolism and detoxification of ROS [109]. However, their specific contributions to sperm function remain inadequately explored. These organelles actively participate in the β‐oxidation of very‐long‐chain fatty acids, a process that inherently produces reactive oxygen, nitrogen, and hydrogen peroxide as byproducts [110]. Peroxisomal enzymes, such as catalase, are critical in regulating hydrogen peroxide levels within the cell. Failure to adequately control hydrogen peroxide can lead to oxidative damage, compromising sperm integrity and function [111, 112].

Endoplasmic reticulum (ER) stress in sperm cells can lead to the generation of ROS, particularly through the disruption of calcium homeostasis and the unfolded protein response [113]. This can exacerbate OS, leading to impaired sperm function. The relationship between ER stress, male infertility, and ROS has been explored by Cao & Kaufman [114], suggesting that ER‐mediated OS might play a role in sperm quality decline.

Leydig and Sertoli cells within the testes have roles that can lead to ROS production. Leydig cells, responsible for testosterone synthesis, utilize cholesterol, which can lead to oxidative byproducts during steroidogenesis [115]. Sertoli cells, which support spermatogenesis, also manage oxidative environments by producing antioxidants like glutathione but can contribute to ROS under stress or inflammation [116]. NADPH oxidases, present in these cells, are another source of ROS, involved in redox signaling but can lead to oxidative stress if their activity is dysregulated [117].

### 7.3 Methods for Measuring Oxidative Stress

OS can be evaluated using various methods, encompassing both direct and indirect assays. Direct assays quantify ROS present in semen samples, whereas indirect assays assess overall antioxidant capacity or detect biomarkers indicative of lipid peroxidation [118]. Each technique has specific advantages and limitations. Table 5 is adapted from Agarwal et al. [118] and provides a summarized comparison of commonly used methods for measuring oxidative stress.

**TABLE 5:** Merits and demerits of direct and indirect methods for assessing oxidative stress in seminal plasma (Adapted from Agarwal et al. [118]).

Assay	Instrumentation	Merits	Demerits
Direct			
Oxidation–reduction potential (ORP)	miOXSYS	Real‐time monitoring of redox balance Timesaving and simple to use Cost‐effective The concentrations of all oxidizing and reducing agents are quantified	The age of a semen sample, its viscosity, and the frequency of centrifugation can impact measurement outcomes
Chemiluminescence	Luminometer Microplate reader (with chemiluminescence detection)	Non‐invasive High sensitivity Real‐time measurement Simple to use	No spatial resolution Susceptibility to background noise that is, Expensive equipment
Indirect			
Malondialdehyde (MDA)	High‐performance liquid chromatography (HPLC) Colorimeter	Measuring lipid peroxidation	Limited sensitivity Non‐specificity Impact of sample preparation
Total antioxidant capacity (TAC)	Colorimeter Luminometer	Simplicity and speed Non‐specific Comprehensive measure of antioxidant potential	Lack of specificity Potential for overestimation Cost of microplate readers
Reactive oxygen species‐Total antioxidant capacity (ROS‐TAC)	Statistical analysis	Improved sensitivity compared to TAC or ROS alone	Complexity and cost Influence of sample matrix Non‐specificity

## 8 Seminal Plasma and Antioxidant Therapy

Seminal plasma plays a significant role in male fertility, not only by acting as a source of transport medium for spermatozoa but also by protecting them from OS [119]. This protection is vital because the male reproductive system is inherently exposed to ROS that can adversely alter sperm motility, morphology, and overall count [105]. To mitigate these detrimental effects, seminal plasma contains a complex antioxidant defense system, which can be classified into enzymatic and non‐enzymatic components [120].

Enzymatic antioxidants such as catalase, glutathione peroxidase (GPx), and superoxide dismutase (SOD) are prominent in the semen [121]. SOD converts superoxide radicals into hydrogen peroxide. Subsequently, catalase and GPx further decompose hydrogen peroxide into water and oxygen. Together, these enzymes synergistically mitigate the removal of oxidants [122].

Beyond enzymatic systems, non‐enzymatic antioxidants such as vitamins (C and E), trace elements (selenium, zinc, chromium, copper), coenzymes (carnitine and Q10), and GSH play a pivotal role in maintaining normal sperm physiology [123]. Vitamin C is a water‐soluble antioxidant that acts as a potent reducing agent that can scavenge free radicals, thereby inhibiting ROS [124]. Vitamin E (tocopherol) is a fat‐soluble antioxidant that protects cell membranes by interrupting the propagation of lipid peroxidation, thus ensuring sperm viability [125].

Ma et al. [126], conducted a randomized controlled trial (RCT) to compare the efficacy of L‐carnitine versus a combination of Coenzyme Q10 (CoQ10) and Vitamin E. A total of 143 men were enrolled and randomized into two groups: one received L‐carnitine complex (15 g twice daily) and the other received Vitamin E (100 mg) and CoQ10 (10 mg), for 3 months (each thrice daily). The authors reported significant improvements in sperm concentration, progressive motility, and morphology in the L‐carnitine group (*p* < 0.0001 for all), while the CoQ10/Vitamin E group showed significant improvements in motility and morphology (*p* = 0.04 and *p* = 0.03, respectively), but not in sperm concentration (*p* = 0.12). Interestingly, testosterone and LH levels significantly increased in the L‐carnitine group (*p* < 0.0001 and *p* = 0.0004), while only testosterone increased in the control group (*p* = 0.0002).

Zinc is a crucial element that maintains the integrity of sperm cells by stabilizing membranes and proteins against oxidative damage [127]. The interplay between zinc, selenium, and these antioxidant components is essential in maintaining the functional capacity of spermatozoa and ensuring optimal fertility [127].

Alpha‐lipoic acid (ALA) is a potent antioxidant with amphipathic characteristics, which allow it to function in both aqueous and lipid phases within cells [128]. Haghighian et al. [129], conducted a randomized, triple‐blind, placebo‐controlled clinical trial to evaluate the efficacy of alpha‐lipoic acid supplementation on semen parameters in infertile men with idiopathic asthenozoospermia. Forty‐four men were randomized into two groups: one received 600 mg ALA daily (*n* = 23), and the other received a matching placebo (*n* = 21) for 12 weeks. The authors reported significant improvements in total sperm count (90.43 ± 6.25 vs. 77.59 ± 4.56 × 10^6^/ejaculate, *p* < 0.001), sperm concentration (26.35 ± 3.17 vs. 22.89 ± 2.74 × 10^6^/mL, *p* < 0.001), and progressive motility (33.48 ± 2.91 vs. 27.14% ± 2.36%, *p* < 0.001) in the ALA group compared to placebo.

Metformin is a widely prescribed antihyperglycemic agent for T2DM with notable antioxidant properties [130]. Beyond its primary purpose of regulating blood glucose levels, metformin has been shown to mitigate OS, thereby enhancing the quality of sperm parameters, reducing lipid peroxidation, and restoring normal levels of pituitary‐gonadal hormones in diabetic individuals [131]. A study by Leisegang et al. [132] reported that metformin administration in diabetes‐induced Wistar rats significantly increased testosterone levels and improved sperm parameters, including motility and viability. This effect was attributed to metformin's ability to reduce lipid peroxidation, oxidative damage, restore normal levels of pituitary‐gonadal hormones, and enhance 5′‐AMP‐activated protein kinase (AMPK) activity [133].

Resveratrol is a polyphenol known for its anti‐inflammatory and antioxidant properties. Evidence from diabetic rat models highlighted its protective effects by attenuating oxidative damage in sperm cells showing decreased markers of lipid peroxidation and increased antioxidant enzyme activity [134]. Additionally, Mongioì et al. [135], showed that resveratrol played a role in safeguarding sperm DNA from oxidative stress. Curcumin has also demonstrated the potential to mitigate oxidative stress, evidenced by reduced apoptotic germ cell death [136] and improved sperm parameters in diabetic rats [137].

### 8.1 Strategies for Managing Oxidative Stress in Diabetic Men

Managing oxidative stress in diabetic men requires an integrated approach that considers the complex interplay of hyperglycemia, obesity, hormonal imbalances, and vascular complications.

Incorporating a diet abundant in antioxidants can significantly bolster the body's intrinsic antioxidant defense mechanism, thereby counteracting OS [138]. Foods high in vitamins C and E, selenium, and flavonoids can help neutralize ROS. Moreover, dietary sources rich in omega‐3 fatty acids, including fish, have been shown to exert anti‐inflammatory effects [139], which contribute to maintaining optimal sperm health by reducing inflammation and oxidative damage to sperm membranes [140].

Regular moderate exercise can promote weight loss, enhance insulin sensitivity, and attenuate oxidative stress, thereby exerting beneficial effects on sperm parameters [141]. However, excessive or unaccustomed exercise might lead to increased ROS production due to increased oxygen consumption [142].

Obesity is associated with elevated OS and diminished fertility potential [143]. Implementing weight loss strategies through dietary modifications and regular physical activity can help reduce systemic inflammation, enhance quantitative and qualitative sperm characteristics [144]. A study by Santi et al. [145] found that weight loss was linked to a reduction in sperm DNA fragmentation, alongside significant improvements in progressive motility and sperm concentration.

Chronic stress elevates cortisol, which can adversely impact the HPG axis, thereby disrupting the regulation of reproductive hormones and potentially impairing male fertility [146]. Techniques like cognitive‐behavioral therapy, yoga, and meditation can mitigate stress, potentially improving fertility outcomes [147].

Excessive alcohol consumption, smoking, and exposure to environmental pollutants are well‐established factors that significantly increase ROS production [148]. Mitigating exposure to these risk factors has been shown to reduce OS, thereby enhancing sperm quality and improving reproductive health outcomes [149].

Reducing the duration and frequency of sauna sessions can help maintain a more favorable thermal environment for sperm production, thereby decreasing OS [150]. Wearing light clothes should become a daily habit, especially for those diagnosed with varicocele, where thermal regulation is already compromised [151].

Managing varicocele encompasses various approaches, including surgical intervention via open or microsurgical techniques, laparoscopic procedures, and percutaneous embolization of the internal spermatic vein [152]. The primary objective of treatment is the occlusion of the pathological venous dilations responsible for testicular drainage abnormalities. Among these modalities, percutaneous embolization is a minimally invasive option that facilitates expedited recovery and demonstrates a procedural success rate of approximately 90% [153].

Due to the high prevalence of functional hypogonadism among diabetic men, comprehensive hormonal profiling, including testosterone, FSH, and LH, should be an integral part of fertility evaluation [154]. While testosterone replacement therapy may be beneficial in selected cases, it must be approached with caution due to its complex and potentially adverse effects on spermatogenesis [155].

### 8.2 Future Perspectives in the Management of Diabetes‐Associated Male Infertility

Management of diabetes‐associated male infertility requires a shift from symptomatic treatment toward strategies that address the underlying metabolic and oxidative mechanisms. Although current interventions such as antioxidant supplementation, lifestyle modification, and optimal glycaemic control have demonstrated benefit, their effects are often partial and inconsistent [156, 157]. Future progress will depend on an integrated approach that combines early detection, precision therapy, and translational research.

Emerging evidence supports the value of comprehensive reproductive screening in men with diabetes, including hormonal assessment, semen analysis, and oxidative stress profiling, even in the absence of overt infertility [158, 159]. Early identification of subclinical reproductive impairment could allow for timely interventions before irreversible testicular damage occurs. This proactive approach may be further strengthened by the incorporation of oxidative stress biomarkers into routine diabetes care, enabling clinicians to monitor the redox status alongside glycaemic control.

Advances in antioxidant therapy also warrant exploration. Novel compounds with dual roles in free radical scavenging and mitochondrial protection, alongside improved formulations of established agents such as coenzyme Q10, alpha‐lipoic acid, and resveratrol, could enhance bioavailability and tissue targeting. The development of delivery systems capable of concentrating antioxidants within the male reproductive tract offers potential to maximize efficacy while minimizing systemic exposure.

Interventions aimed at restoring testicular microvascular integrity and cellular function represent another promising area. Experimental models suggest that mesenchymal stem cell‐derived factors and exosome‐based therapies may attenuate oxidative damage, support Leydig and Sertoli cell function, and improve spermatogenesis [160, 161, 162]. While clinical translation will require rigorous safety and efficacy trials, these findings highlight the potential for regenerative strategies in selected patient populations.

Lifestyle optimization will remain central to any management plan. Sustained weight reduction, dietary patterns enriched in natural antioxidants, and structured physical activity have consistently been shown to improve both metabolic health and semen parameters [163, 164, 165]. Digital health technologies, including continuous glucose monitoring and app‐based fertility tracking, may support adherence by providing real‐time feedback and integrating metabolic and reproductive health goals.

Ultimately, improving fertility outcomes in diabetic men will require multidisciplinary collaboration between relevant health care professionals like endocrinologists, urologists, reproductive biologists, nutritionists, and mental health specialists. Long‐term, multicenter clinical studies are needed to define optimal combinations of pharmacological, nutritional, and behavioral interventions. Developing standardized protocols to integrate reproductive health considerations into diabetes management is also crucial for enhancing fertility outcomes in men with diabetes.

## 9 Conclusion

Diabetes mellitus exerts a profound and multifaceted impact on male fertility, with oxidative stress emerging as a central mechanism. Hyperglycemia‐induced activation of pathways such as the polyol pathway and AGEs formation depletes antioxidant defenses and promotes reactive oxygen species overproduction, culminating in structural, functional, and genetic damage to spermatozoa. The resulting impairments in semen quality, hormonal regulation, and testicular function translate into reduced natural conception rates and poorer assisted reproduction outcomes. Compounding factors such as obesity and metabolic syndrome further intensify oxidative damage, underscoring the complexity of managing fertility in diabetic men. Current evidence highlights the need for integrated approaches that combine stringent glycemic control, targeted antioxidant therapy, and lifestyle interventions to mitigate oxidative injury. Advancing research into novel therapeutics and early screening strategies will be critical in preserving reproductive potential and improving the quality of life for men affected by diabetes.

## Author Contributions

S.M.D., A.O.A., and K.M. contributed to study conceptualization, manuscript drafting, and table creation. C.S.O. provided conceptualization, manuscript review, and supervision. B.C.L. contributed manuscript editing and supervision.

## Ethics Statement

The authors have nothing to report.

## Conflicts of Interest

The authors declare no conflicts of interest.
